# The paradoxical functions of EGFR during breast cancer progression

**DOI:** 10.1038/sigtrans.2016.42

**Published:** 2017-01-20

**Authors:** Remah Ali, Michael K Wendt

**Affiliations:** 1 Department of Medicinal Chemistry and Molecular Pharmacology, Purdue University Center for Cancer Research, Purdue University, West Lafayette, IN, USA

## Abstract

The epidermal growth factor receptor (EGFR) is one of the most well-studied signaling pathways in cancer progression. As a result, numerous therapeutics including small-molecule inhibitors and monoclonal antibodies have been developed to target this critical oncogenic driver. Several of these EGFR inhibitors (EGFRi) have been evaluated in metastatic breast cancer, as high-level EGFR expression in primary tumors correlates with the highly aggressive basal-like phenotype and predicts for poor patient prognosis. Surprisingly, these trials have been unanimously unsuccessful at improving patient outcomes. Numerous factors, such as lack of proper patient selection may have contributed to the failure of these trials. However, recent findings suggest that there are fundamental changes in EGFR signaling that take place during primary tumor invasion, dissemination and ultimate metastasis of breast cancer cells. Herein, we review the outcomes of EGFR-targeted clinical trials in breast cancer and explore our current understanding of EGFR signaling within primary mammary tumors and how these events are altered in the metastatic setting. Overall, we put forth the hypothesis that fundamental changes in EGFR signaling between primary and metastatic tumors, a process we term the ‘EGFR paradox,’ contribute to the clinically observed inherent resistance to EGFRi. Furthermore, this hypothesis introduces the possibility of utilizing EGFR agonism as a potential therapeutic approach for the treatment of metastatic breast cancer.

## Introduction

**Figure 1:**
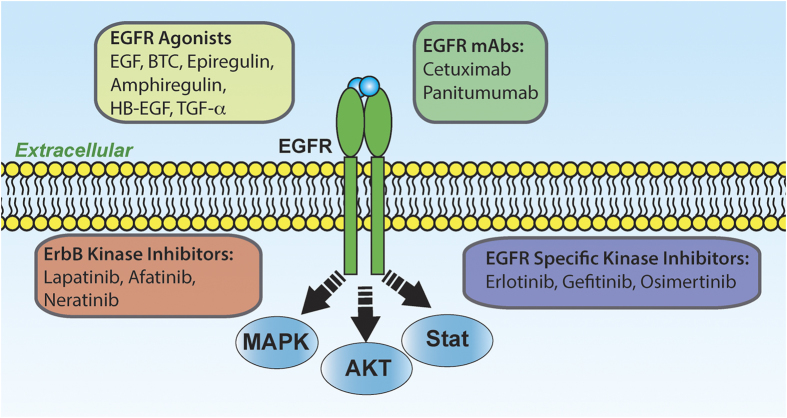
A schematic representation of the activators, inhibitors and outcomes of EGFR signaling. EGFR is part of the four-member ErbB superfamily (ErbB1–4). These receptors form several different homo- and heterodimers (here we only depict the EGFR homodimer). EGFR is capable of binding several different extracellular ligands that agonize the receptor leading to activation of several downstream signaling events including, but no limited to those listed. Several therapeutics have been developed to antagonize EGFR including monoclonal antibodies (mAbs) that block ligand binding as well as several different kinase inhibitors. In addition to EGFR, some of these kinase inhibitors also target other ErbB receptors, supporting their use in Her2-amplified BC. All of the listed therapies are FDA approved for various cancers with the exception of Neratinib.

Epidermal growth factor receptor (EGFR) was the first discovered of the ErbB family of receptor tyrosine kinases which includes a total of four members: Erbb1/EGFR, ErbB2/Her2, ErbB3 and ErbB4.^1^ ErbB members form homo- and heterodimeric cell-surface receptors with unique extracellular domains yielding ligand-binding specificity. Downstream signaling from these receptors proceeds via tyrosine phosphorylation.^2^ Since its discovery, EGFR has been characterized as a mediator of a wide variety of signal transduction events that control cell proliferation, migration and survival. Overexpression of EGFR transforms NIH3T3 fibroblasts in an EGF-dependent manner.^3^ Aberrant EGFR activation in tumor cells can result from increased transcriptional expression and/or gene amplification. Increased EGFR protein and transcript levels correlate with poor prognosis in various epithelial cancers, such as colorectal cancer (CRC),^4^ non-small cell lung cancer (NSCLC),^5^ endometrial cancer,^6^ and squamous-cell carcinoma of the head and neck (SCCHN).^7^ Another mode of EGFR activation in cancer is activating somatic mutations that result in constitutive kinase activity, and these are particularly prevalent in NSCLC (reviewed in Morgensztern *et al.* ^8^). These findings have led to the development of numerous FDA-approved EGFR inhibitors for many of these cancers (Figure 1). Gefitinib is a small molecule EGFR kinase inhibitor that received accelerated approval from the FDA in 2003 but was pulled from the market due to lack of efficacy. These findings were the result of not selecting patients whose tumors contain EGFR activating mutations. Since then, it has been recognized that only NSCLC patients with activating mutations in EGFR respond to gefitinib. This led to the 2015 approval of gefitinib as a first-line therapy for NSCLC specifically in patients that test positive for activating EGFR mutations. The addiction of these tumors to EGFR signaling is further demonstrated by the emergence of the secondary activating T790M mutation as a major cause of tumor resistance to gefitinib. This has resulted in the recent formulation and FDA approval of osimertinib, a compound capable of inhibiting T790M mutant EGFR.^9^ These lessons in NSCLC have served as a critical example of the need for biomarkers to drive application of kinase inhibitors to EGFR. Although activating mutations in EGFR are prevalent in NSCLC patients, inhibition of wild-type EGFR has shown success in pancreatic cancer,^10^ head and neck cancer^11^ and colorectal cancer.^12^ Ultimately, these studies have led to the FDA approval of EGFR ligand blocking antibodies (cetuximab and panitumumab) for the treatment of colorectal and head and neck cancers. However, studies are still ongoing to determine other biomarkers that might improve patient selection for these cancers.^13^

## Targeting EGFR in metastatic breast cancer

Breast cancer (BC) is the most commonly diagnosed and the second most lethal cancer in American women.^14^ Metastasis is invariably responsible for patient death in BC. The triple negative BC subtype (TNBC) is characterized by metastatic progression, poor patient prognosis, and is identified by the absence of bio-molecules that form the basis for targeted therapies for the other BC subtypes, namely estrogen receptor, progesterone receptor, and Her2 amplification.^15^ Thus, there are currently no FDA approved targeted therapies for TNBC. TNBC is initially highly sensitive to chemotherapy, but many TNBC patients rapidly develop resistance, at which point metastatic disease is highly lethal.^16^ Although activating mutations and gene amplification of EGFR are rare in BC, EGFR expression can be enhanced by increased gene copy number due to polysomy, and enhanced expression of EGFR in the primary tumor is associated with increased metastasis and decreased survival of TNBC patients.^17,18^ Concomitant with these clinical findings, studies from the Condeelis lab established a paracrine signaling loop in which macrophage-produced EGF supported tumor cell invasion and dissemination from the primary tumor.^19,20^ Experimental findings such as these prompted the initiation of several clinical trials to assess the effectiveness of EGFR inhibition (EGFRi) in metastatic TNBC. The EGFR kinase inhibitor erlotinib was evaluated in a phase II trial of unselected patients with advanced BC having had previously received chemotherapy.^21^ In addition, erlotinib was evaluated in combination with the anti-vascular endothelial growth factor (VEGF) antibody bevacizumab.^22^ Both of these studies determined that erlotinib did not provide clinical benefit to BC patients and erlotinib responsiveness was not predicted by EGFR expression levels in the primary tumor.

Gefitinib is another EGFR-specific kinase inhibitor that has been evaluated in metastatic BC in multiple trials. A multicenter phase II study examined the outcomes of gefitinib treatment in unselected metastatic BC patients that had previously received standard chemotherapies. In all, 98.3% of these patients were non-responders and as above there was no correlation between EGFR expression and response to gefitinib.^23^ Similarly, gefitinib as a monotherpay in metastatic estrogen receptor alpha (ER-α) negative BC patients did not provide clinical benefit in another phase II clinical trial.^24^ Engebraaten *et al.* tested the efficacy of combining gefitinib with docetaxel in metastatic BC as compared with docetaxel alone. In this study, the combination was associated with lower partial response rate and higher toxicity than chemotherapy alone.^25^ In addition to kinase inhibitors, clinical trials have also evaluated the addition of the ligand blocking monoclonal antibody cetuximab to the DNA-alkylating agent carboplatin.^26^ Similarly, this study found that fewer than 20% of metastatic TNBC patients responded to cetuximab plus carboplatin. In subsequent studies, the combination of cetuximab with antimicrotubule agents or topoisomerase inhibitors did not increase patient overall survival as compared with these chemotherapies alone, leading to premature trial termination.^27,28^ These findings have been confirmed in more recent trials examining the efficacy of panitumumab, another ligand-blocking anti-EGFR monoclonal antibody, in the treatment of TNBC. As with other EGFRi, panitumumab did not improve progression-free survival over chemotherapy alone when used in metastatic TNBC.^29^ In contrast to these adjuvant trials in metastatic disease, use of panitumumab in combination with chemotherapy did appear efficacious as a neoadjuvant therapy for operable stage II–III TNBC.^30^ Overall, despite strong pre-clinical data linking high levels of EGFR to increased metastatic progression and decreased patient survival, TNBC in the metastatic setting appears to be unresponsive to EGFRi (Table 1). The mechanisms of inherent resistance of metastatic BC to EGFRi remain to be fully established.

## The ‘EGFR paradox’ during the metastatic progression of breast cancer

Recently, our lab reported findings that demonstrate a switch in EGFR function between primary and metastatic tumors.^31^ In this study, EGF treatment of EGFR-amplified primary tumor cells resulted in increased proliferation, and these cells were particularly sensitive to EGFR inhibition. Conversely, after epithelial–mesenchymal transition (EMT)-driven *in vivo* metastasis, cells derived from pulmonary metastases are inherently resistant to EGFRi and undergo robust growth inhibition in response to EGF.^31^ This idea that growth factors have context dependent dual effects on cell growth has long been proposed.^32^ Indeed, growth factors such as interleukin 6 (IL-6) and platelet-derived growth factor (PDGF) are known to paradoxically inhibit the growth of some cell types.^33,34^ Furthermore, the recognized growth-promoting roles of estrogen in BC are coupled with accounts of estrogen-induced apoptosis, termed ‘the estrogen paradox’ nicely reviewed in Jordan and Ford.^35^ Another well-established shift in function in BC is that of transforming growth factor-beta (TGF-β) where it functions as a powerful tumor suppressor in primary tumors but drives disease progression in the metastatic setting.^36^ Further understanding of this shift in EGFR signaling will likely serve to explain the failure of EGFRi in the treatment of metastatic BC. Furthermore, these findings also present the opportunity to exploit the antimetastatic function of EGFR agonism as a therapeutic approach. Below we review some of the established findings that support the existence of the EGFR paradox during BC growth, dissemination and metastasis.

## Potential mechanisms of inherent resistance to EGFRi in metastatic breast cancer

### Diminution of EGFR expression with metastatic progression

As mentioned above, our lab recently developed a model in which overexpression of WT EGFR transforms normal murine mammary gland (NMuMG) cells.^31,37–39^ This EGFR-driven tumor model forms well-differentiated *in situ* mammary tumors, but following induction of EMT metastatic tumors derived from these same cells demonstrate reduced expression of EGFR and inherent resistance to erlotinib.^31^ Similarly, *in vivo* metastatic selection of the heterogeneous MDA-MB-231 TNBC cells is associated with a marked loss of EGFR expression.^31^ This discordance in EGFR expression is observed clinically and in mouse models of metastatic colorectal cancer,^40^ ovarian cancer^41^ and lung cancer.^42^ The first observation that metastatic BC cells can have low to undetectable levels of EGFR was reported for the DU4475 (cutaneous metastasis) and AlAb 496 (lung metastasis) cell models in 1982.^43^ Since then, isogenic BC cell-line series have demonstrated EGFR downregulation through metastatic progression, including the MCF10AT BC progression series and the D2-HAN series.^44–47^ In patient-derived BC tissues, EGFR is downregulated with metastasis and this correlates with resistance to EGFR inhibitors.^44,48^ Similarly, EGFR downregulation through promoter hyper-methylation has been linked to inherent resistance to anti-EGFR therapy in colorectal carcinoma.^49^ In BC, however, the mechanism(s) of EGFR attenuation that are responsible for metastatic resistance to EGFRi remain largely unknown.

### EGFR enhanced nuclear transport after metastasis

EGFR is primarily localized to the plasma membrane, but numerous studies have demonstrated nuclear localization of EGFR where it can undergo several poorly understood functions that are both dependent and independent of kinase activity.^50–52^ One of the seminal studies reporting EGFR nuclear translocation was done by Lin *et al.*,^53^ who described the nuclear function of EGFR as a transcription factor, and established its endogenous target genes, and consensus DNA-binding sequence. Readers seeking an in-depth review on the transport mechanisms and functions of nuclear EGFR are referred to the following.^51^ Importantly, increased nuclear transport of EGFR has been suggested as a potential mechanism of acquired resistance to EGFRi. This was shown in studies demonstrating that long-term treatment of a NSCLC cell line with cetuximab generates cell clones that have enhanced nuclear EGFR staining.^54^ Similarly in BC, nuclear EGFR has been attributed to inherent resistance to cetuximab and gefitinib using various TNBC cell lines.^55,56^ Retrospective studies using patient-derived samples linking enhanced nuclear EGFR to clinical EGFRi resistance are yet to be performed. These investigations will be essential to confirm the role of nuclear EGFR in resistance to EGFRi therapy. If differential subcellular localization of EGFR is truly at play during inherent resistance to EGFRi, establishing small-molecule inhibitors that specifically localize to these compartments will be essential to understanding and targeting this mechanism in metastatic BC.^57^

### The growth-inhibitory function of EGFR

The first observation that EGF inhibits cancer-cell growth at concentrations that are stimulatory to other cells was reported for the rat pituitary GH4CI tumor cell line and the human epidermoid carcinoma A431 cell line.^58–60^ EGF inhibition of growth has also been demonstrated for human BC cell lines, where higher concentrations of EGF decreased DNA synthesis in MCF-7, SK-Br-3, BT-20, BT-474 cells.^43^ MDA-MB-468 is an EGFR amplified BC cell line derived from a pleural effusion that is also known to display marked EGF-induced growth inhibition due to induction of apoptosis.^61,62^ The A431 and MDA-MB-468 cell lines have abnormally high levels of EGFR, and therefore the idea has been purported that the receptor must be present above a critical threshold to induce growth inhibition.^61,63^ However, this does not seem to be solely responsible for this phenomenon as EGF-induced inhibition of cell-growth occurs in various non-EGFR amplified cell-lines.^43,64^ Further, EGF treatment stimulates the growth of several BC cell lines expressing extremely high levels of EGFR.^3,31,65^ Overall, the strongest body of literature supports that the growth-inhibitory action of EGF is largely due to induction of apoptosis. The mechanisms of EGF-induced apoptosis are still not fully understood, but seem to involve signaling events that take place following receptor internalization potentially resulting in endosomal accumulation.^66,67^ In addition, EGFR-mediated activation of signal transducer and activator of transcription-1 (STAT1) has been shown to induce apoptosis via activation of caspases, induction of elements of the interferon pathway, or by mediating cell cycle arrest by activation of p21.^68–73^

### The impact of co-expressed receptors on resistance to EGFR kinase inhibitors

The above mechanisms of inherent resistance to EGFRi dictate the emergence of alternative signaling pathways that sustain tumor cell survival and metastatic growth. In esophageal cancer, resistance to EGFRi is associated with fibroblast growth factor receptor 2 (FGFR2) amplification and overexpression.^74^ In NSCLC, the amplification of hepatocyte growth factor receptor (c-MET) has been implicated in resistance to EGFR kinase inhibition by reactivating ErbB signaling through the ErbB3 receptor.^75^ Studies in NSCLC also suggest the Axl receptor can facilitate resistance to erlotinib.^76^ Similarly, Axl has been found to interact with and transactivate signaling from EGFR and other receptors independent of ligand engagement in TNBC cells.^77^ Clearly this mechanism would contribute to resistance to ligand-blocking EGFR antibodies. In our EGFR-driven metastatic BC model, the diminution and functional switch of EGFR in metastatic lesions is associated with an increase in fibroblast growth factor receptor 1 splice variant β (FGFR1-β) expression.^39^ Indeed, recent studies from our lab and others highlight the value of FGFR targeting therapies in TNBC and lapatinib-resistant BC models.^78–81^ Interestingly, separate studies point to the role of β3 integrin in mediating NFκB signaling and resistance to erlotinib.^82^ Along these lines, we find that β3 integrin is absolutely required for FGFR signaling in BC.^78^ Further mechanistic understanding of how BC cancer cells upregulate alternative growth pathways to replace the driver function of EGFR will expand the therapeutic options for BC patients in the metastatic setting.

## Discussion and conclusion

**Figure 2:**
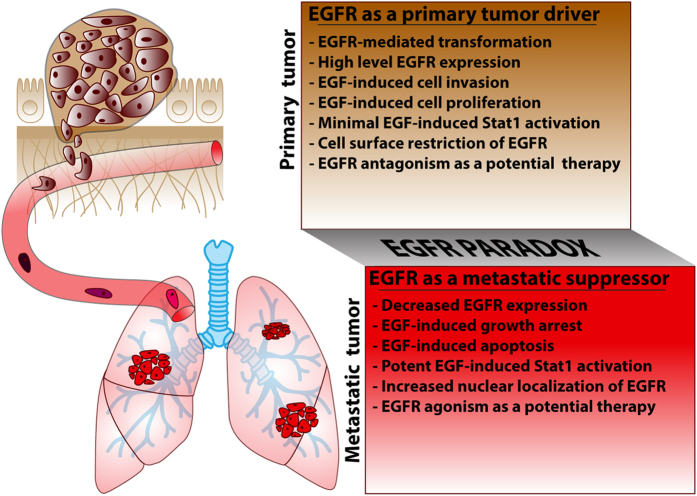
Schematic description of the EGFR paradox in primary versus metastatic BC. As tumor cells invade and disseminate, numerous selective pressures drive fundamental changes in cell signaling and growth versus death stimuli (noted by the changing colors of the tumor cells). These selective pressures and the unique microenvironment of the metastatic destination (depicted here as the lungs) yield metastatic tumors that can be quite diverse from the primary tumor. These events contribute to the listed fundamental changes in EGFR signaling in metastases as compared with primary breast tumors, constituting the ‘EGFR paradox.’ Overall, these events likely contribute to the failure of EGFRi therapies for the treatment of metastatic disease. In addition, these events point to EGFR agonism as a potential therapeutic strategy in metastatic BC.

EGFR is a critical signaling molecule involved in a myriad of biological processes and carcinogenic events. In various cancers, aberrant EGFR activation contributes to the initial oncogenic transformation of cells and their subsequent invasion and exit from the primary tumor. These canonical signaling events generated from plasma membrane localized receptors drive the well-established oncogenic effects of EGFR. In contrast, progression of disseminated BC cells into macrometastases is associated with downregulation of EGFR, shunting of EGFR away from the cell surface, and non-canonical pro-apoptotic signaling through STAT1. Although a precise mechanism that unifies these observations remains unknown, EGFR induction of apoptosis has previously been demonstrated to result from intracellular signaling from endosomes.^66,67^ Thus, it is tempting to speculate a model where cell surface EGFR signaling has a crucial role for the initial invasion of BC.^37^ However, following systemic dissemination, tumor cells will increase EGFR internalization and/or decrease its expression as part of an adaptation response to the metastatic microenvironment. Thus, established macrometastases evolve to become EGFR independent and are therefore inherently resistant to targeted inhibition of EGFR (Figure 2).

### Future challenges of EGFR therapy in BC

Inhibition of EGFR signaling via kinase inhibitors and monoclonal antibodies has resulted in fundamental changes in patient care for some tumor types. However, numerous attempts to apply these therapies to metastatic BC patients have been unsuccessful. We believe the literature as a whole supports a paradoxical shift in EGFR function during BC metastasis to a STAT1-dominated pro-apoptotic signaling mechanism. Other STAT1-activating cytokines are heavily used therapeutically, yet EGFR–STAT1 signaling is virtually unexplored in cancer treatment. We conclude that EGFR agonism could be pursued as a potential adjuvant therapy for metastatic BC. Despite the potential utility of EGFR agonism as a therapeutic approach in metastatic BC, predicting patient groups that might benefit from EGFR agonists versus inhibitors faces many challenges. Paramount to these challenges includes the design of effective biomarkers to predict the pro- versus anti-tumorigenic effect of EGFR. Although EGFR expression and cellular localization can be assessed in primary mammary tumor biopsies, these types of analyses would need to be standardized into reproducible diagnostics that could be introduced to the clinic. Furthermore, these detection methods may not be feasible on metastatic BC tissues.

However, using the estrogen paradox as a model, estrogen treatment has demonstrated growth inhibitory effects on BC cells in culture and in mouse models.^83,84^ Similarly, patients pretreated and resistant to endocrine inhibition therapies do show antitumor responses when switched to high-dose estrogen.^85,86^ Therefore, one potential course of therapy for patients who present with metastatic lesions and display EGFR expression in their primary tumor would be to initiate EGFRi treatment, and at the point of disease progression, abruptly switch to a high-dose EGFR agonist. Indeed, a recent study using the A431 model of EGF-induced growth inhibition has established proof-of-concept for *in vivo* tumor inhibition upon systemic administration of supraphysiologic levels of recombinant EGF.^87^ Overall, more thorough preclinical and clinical studies will establish if we will be able to harness the power of the EGFR paradox for the therapeutic benefit of metastatic BC patients.

## Figures and Tables

**Table 1:** A summary of clinical studies investigating EGFRi therapies for the treatment of breast cancer

*EGFRi class*	*Drug*	*Targeted BC patient group*	*Benefit over control*	*Reference*
EGFR kinase inhibitors	Erlotinib	Phase II of locally advanced or metastatic BC as a monotherapy	No	Dickler *et al.* ^21^
	Erlotinib	Phase II of metastatic BC in combination with anti-VEGF mAb	No	Dickler *et al.* ^22^
	Gefitinib	Phase II of metastatic BC as a monotherapy	No	Minckwitz *et al.* ^23^
	Gefitinib	Phase II of metastatic ER-α negative BC as a monotherapy	No	Green *et al.* ^24^
	Gefitinib	Phase II of metastatic BC in combination with chemotherapy	No	Engebraaten *et al.* ^25^
EGFR ligand-blocking monoclonal antibody (mAb)	Cetuximab	Phase II of metastatic TNBC, in combination with chemotherapy	No	Carey *et al.* ^26^
	Cetuximab	Metastatic TNBC, in combination with chemotherapy	No	Trédan *et al.* ^27^
	Cetuximab	Phase II of metastatic BC in combination with chemotherapy	No	Crozier *et al.* ^28^
	Panitumumab	Phase II of metastatic TNBC, in combination with chemotherapy	No	Yardley *et al.* ^29^
	Panitumumab	Neoadjuvant therapy for operable primary TNBC	Yes	Nabholtz *et al.* ^30^

## References

[bib1] Cohen S , Fava RA , Sawyer ST . Purification and characterization of epidermal growth factor receptor/protein kinase from normal mouse liver. Proc Natl Acad Sci USA 1982; 79: 6237–6241. 6983068 10.1073/pnas.79.20.6237 PMC347095

[bib2] Yarden Y , Sliwkowski MX . Untangling the ErbB signalling network. Nat Rev Mol Cell Biol 2001; 2: 127–137. 11252954 10.1038/35052073

[bib3] Di Fiore PP , Pierce JH , Fleming TP , Hazan R , Ullrich A , King CR et al. Overexpression of the human EGF receptor confers an EGF-dependent transformed phenotype to NIH 3T3 cells. Cell 1987; 51: 1063–1070. 3500791 10.1016/0092-8674(87)90592-7

[bib4] Spano J-P , Lagorce C , Atlan D , Milano G , Domont J , Benamouzig R et al. Impact of EGFR expression on colorectal cancer patient prognosis and survival. Ann Oncol 2005; 16: 102–108. 15598946 10.1093/annonc/mdi006

[bib5] Brabender J , Danenberg KD , Metzger R , Schneider PM , Park J , Salonga D et al. Epidermal growth factor receptor and HER2-neu mRNA expression in non-small cell lung cancer is correlated with survival. Clin Cancer Res 2001; 7: 1850–1855. 11448895

[bib6] Konecny GE , Santos L , Winterhoff B , Hatmal M , Keeney GL , Mariani A et al. HER2 gene amplification and EGFR expression in a large cohort of surgically staged patients with nonendometrioid (type II) endometrial cancer. Br J Cancer 2009; 100: 89–95. 19088718 10.1038/sj.bjc.6604814 PMC2634683

[bib7] Rubin Grandis J , Melhem MF , Barnes EL , Tweardy DJ . Quantitative immunohistochemical analysis of transforming growth factor-alpha and epidermal growth factor receptor in patients with squamous cell carcinoma of the head and neck. Cancer 1996; 78: 1284–1292. 8826952 10.1002/(SICI)1097-0142(19960915)78:6<1284::AID-CNCR17>3.0.CO;2-X

[bib8] Morgensztern D , Politi K , Herbst RS . Egfr mutations in non-small-cell lung cancer: find, divide, and conquer. JAMA Oncol 2015; 1: 146–148. 26181013 10.1001/jamaoncol.2014.278

[bib9] Jänne PA , Yang JC-H , Kim D-W , Planchard D , Ohe Y , Ramalingam SS et al. AZD9291 in EGFR inhibitor-resistant non-small-cell lung cancer. N Engl J Med 2015; 372: 1689–1699. 25923549 10.1056/NEJMoa1411817

[bib10] Moore MJ , Goldstein D , Hamm J , Figer A , Hecht JR , Gallinger S et al. Erlotinib plus gemcitabine compared with gemcitabine alone in patients with advanced pancreatic cancer: a phase III trial of the National Cancer Institute of Canada Clinical Trials Group. J Clin Oncol 2007; 25: 1960–1966. 17452677 10.1200/JCO.2006.07.9525

[bib11] Vermorken JB , Mesia R , Rivera F , Remenar E , Kawecki A , Rottey S et al. Platinum-based chemotherapy plus cetuximab in head and neck cancer. N Engl J Med 2008; 359: 1116–1127. 18784101 10.1056/NEJMoa0802656

[bib12] Van Cutsem E , Peeters M , Siena S , Humblet Y , Hendlisz A , Neyns B et al. Open-label phase III trial of panitumumab plus best supportive care compared with best supportive care alone in patients with chemotherapy-refractory metastatic colorectal cancer. J Clin Oncol 2007; 25: 1658–1664. 17470858 10.1200/JCO.2006.08.1620

[bib13] Sunakawa Y , Yang D , Moran M , Astrow SH , Tsuji A , Stephens C et al. Combined assessment of EGFR-related molecules to predict outcome of 1st-line cetuximab-containing chemotherapy for metastatic colorectal cancer. Cancer Biol Ther 2016; 17: 751–759. 27104867 10.1080/15384047.2016.1178426 PMC4970538

[bib14] American Cancer Society. American Cancer Society, Cancer Treatment and Survivorship Facts and Figures, 2016. Available at http://www.cancer.org/acs/groups/content/@research/documents/document/acspc-047079.pdf.

[bib15] Lehmann BD , Pietenpol JA , Tan AR . Triple-negative breast cancer: molecular subtypes and new targets for therapy. Am Soc Clin Oncol Educ Book 2015; 35: e31–e39. 10.14694/EdBook_AM.2015.35.e31 25993190

[bib16] Uhm JE , Park YH , Yi SY , Cho EY , Choi YL , Lee SJ et al. Treatment outcomes and clinicopathologic characteristics of triple-negative breast cancer patients who received platinum-containing chemotherapy. Int J Cancer 2009; 124: 1457–1462. 19065658 10.1002/ijc.24090

[bib17] Park HS , Jang MH , Kim EJ , Kim HJ , Lee HJ , Kim YJ et al. High EGFR gene copy number predicts poor outcome in triple-negative breast cancer. Mod Pathol 2014; 27: 1212–1222. 24406864 10.1038/modpathol.2013.251

[bib18] Tischkowitz M , Brunet J-S , Bégin LR , Huntsman DG , Cheang MCU , Akslen LA et al. Use of immunohistochemical markers can refine prognosis in triple negative breast cancer. BMC Cancer 2007; 7: 134. 17650314 10.1186/1471-2407-7-134 PMC1948892

[bib19] Wyckoff J , Wang W , Lin EY , Wang Y , Pixley F , Stanley ER et al. A paracrine loop between tumor cells and macrophages is required for tumor cell migration in mammary tumors. Cancer Res 2004; 64: 7022–7029. 15466195 10.1158/0008-5472.CAN-04-1449

[bib20] Patsialou A , Wyckoff J , Wang Y , Goswami S , Stanley ER , Condeelis JS . Invasion of human breast cancer cells *in vivo* requires both paracrine and autocrine loops involving the colony-stimulating factor-1 receptor. Cancer Res 2009; 69: 9498–9506. 19934330 10.1158/0008-5472.CAN-09-1868 PMC2794986

[bib21] Dickler MN , Cobleigh MA , Miller KD , Klein PM , Winer EP . Efficacy and safety of erlotinib in patients with locally advanced or metastatic breast cancer. Breast Cancer Res Treat 2009; 115: 115–121. 18496750 10.1007/s10549-008-0055-9

[bib22] Dickler MN , Rugo HS , Eberle CA , Brogi E , Caravelli JF , Panageas KS et al. A phase II trial of erlotinib in combination with bevacizumab in patients with metastatic breast cancer. Clin Cancer Res 2008; 14: 7878–7883. 19047117 10.1158/1078-0432.CCR-08-0141 PMC2748748

[bib23] von Minckwitz G , Jonat W , Fasching P , Bois A , du, Kleeberg U , Lück H-J et al. A multicentre phase II study on gefitinib in taxane- and anthracycline-pretreated metastatic breast cancer. Breast Cancer Res Treat 2005; 89: 165–172. 15692759 10.1007/s10549-004-1720-2

[bib24] Green MD , Francis PA , Gebski V , Harvey V , Karapetis C , Chan A et al. Gefitinib treatment in hormone-resistant and hormone receptor-negative advanced breast cancer. Ann Oncol 2009; 20: 1813–1817. 19553291 10.1093/annonc/mdp202

[bib25] Engebraaten O , Edvardsen H , Løkkevik E , Naume B , Kristensen V , Ottestad L et al. Gefitinib in combination with weekly docetaxel in patients with metastatic breast cancer caused unexpected toxicity: results from a randomized phase II clinical trial. ISRN Oncol 2012; 2012: 176789. 22666610 10.5402/2012/176789 PMC3361199

[bib26] Carey LA , Rugo HS , Marcom PK , Mayer EL , Esteva FJ , Ma CX et al. TBCRC 001: randomized phase II study of cetuximab in combination with carboplatin in stage IV triple-negative breast cancer. J Clin Oncol 2012; 30: 2615–2623. 22665533 10.1200/JCO.2010.34.5579 PMC3413275

[bib27] Trédan O , Campone M , Jassem J , Vyzula R , Coudert B , Pacilio C et al. Ixabepilone alone or with cetuximab as first-line treatment for advanced/metastatic triple-negative breast cancer. Clin Breast Cancer 2015; 15: 8–15. 25218708 10.1016/j.clbc.2014.07.007

[bib28] Crozier JA , Advani PP , LaPlant B , Hobday T , Jaslowski AJ , Moreno-Aspitia A et al. N0436 (alliance): a phase II trial of irinotecan with cetuximab in patients with metastatic breast cancer previously exposed to anthracycline and/or taxane-containing therapy. Clin Breast Cancer 2016; 16: 23–30. 26381420 10.1016/j.clbc.2015.08.002 PMC4698217

[bib29] Yardley DA , Ward PJ , Daniel BR , Eakle JF , Lamar RE , Lane CM et al. Panitumumab, gemcitabine, and carboplatin as treatment for women with metastatic triple-negative breast cancer: a Sarah Cannon Research Institute phase II trial. Clin Breast Cancer 2016; 16: 349–355. 27340049 10.1016/j.clbc.2016.05.006

[bib30] Nabholtz JM , Abrial C , Mouret-Reynier MA , Dauplat MM , Weber B , Gligorov J et al. Multicentric neoadjuvant phase II study of panitumumab combined with an anthracycline/taxane-based chemotherapy in operable triple-negative breast cancer: identification of biologically defined signatures predicting treatment impact. Ann Oncol 2014; 25: 1570–1577. 24827135 10.1093/annonc/mdu183

[bib31] Wendt MK , Williams WK , Pascuzzi PE , Balanis NG , Schiemann BJ , Carlin CR et al. The antitumorigenic function of EGFR in metastatic breast cancer is regulated by expression of Mig6. Neoplasia 2015; 17: 124–133. 25622905 10.1016/j.neo.2014.11.009 PMC4309683

[bib32] Sporn MB , Roberts AB . Peptide growth factors are multifunctional. Nature 1988; 332: 217–219. 2831460 10.1038/332217a0

[bib33] Minami M , Inoue M , Wei S , Takeda K , Matsumoto M , Kishimoto T et al. STAT3 activation is a critical step in gp130-mediated terminal differentiation and growth arrest of a myeloid cell line. Proc Natl Acad Sci USA 1996; 93: 3963–3966. 8632998 10.1073/pnas.93.9.3963 PMC39468

[bib34] Kim HR , Upadhyay S , Li G , Palmer KC , Deuel TF . Platelet-derived growth factor induces apoptosis in growth-arrested murine fibroblasts. Proc Natl Acad Sci USA 1995; 92: 9500–9504. 7568162 10.1073/pnas.92.21.9500 PMC40829

[bib35] Jordan VC , Ford LG . Paradoxical clinical effect of estrogen on breast cancer risk: a ‘new’ biology of estrogen-induced apoptosis. Cancer Prev Res (Phila) 2011; 4: 633–637. 21478501 10.1158/1940-6207.CAPR-11-0185 PMC3100896

[bib36] Tian M , Schiemann WP . The TGF-beta paradox in human cancer: an update. Future Oncol Lond Engl 2009; 5: 259–271. 10.2217/14796694.5.2.259 PMC2710615 19284383

[bib37] Wendt MK , Smith JA , Schiemann WP . Transforming growth factor-β-induced epithelial-mesenchymal transition facilitates epidermal growth factor-dependent breast cancer progression. Oncogene 2010; 29: 6485–6498. 20802523 10.1038/onc.2010.377 PMC3076082

[bib38] Wendt MK , Balanis N , Carlin CR , Schiemann WP . STAT3 and epithelial-mesenchymal transitions in carcinomas. JAK-STAT 2014; 3: e28975. 24843831 10.4161/jkst.28975 PMC4024059

[bib39] Wendt MK , Taylor MA , Schiemann BJ , Sossey-Alaoui K , Schiemann WP . Fibroblast growth factor receptor splice variants are stable markers of oncogenic transforming growth factor β1 signaling in metastatic breast cancers. Breast Cancer Res 2014; 16: R24. 24618085 10.1186/bcr3623 PMC4053226

[bib40] Scartozzi M , Bearzi I , Mandolesi A , Galizia E , Pierantoni C , Loupakis F et al. Epidermal growth factor receptor gene promoter methylation in primary colorectal tumors and corresponding metastatic sites: a new perspective for an ‘old’ therapeutic target. Anal Quant Cytol Histol 2009; 31: 417–423. 20698358

[bib41] Pradeep S , Kim SW , Wu SY , Nishimura M , Chaluvally-Raghavan P , Miyake T et al. Hematogenous metastasis of ovarian cancer: rethinking mode of spread. Cancer Cell 2014; 26: 77–91. 25026212 10.1016/j.ccr.2014.05.002 PMC4100212

[bib42] Zohrabian VM , Nandu H , Gulati N , Khitrov G , Zhao C , Mohan A et al. Gene expression profiling of metastatic brain cancer. Oncol Rep 2007; 18: 321–328. 17611651

[bib43] Imai Y , Leung CK , Friesen HG , Shiu RP . Epidermal growth factor receptors and effect of epidermal growth factor on growth of human breast cancer cells in long-term tissue culture. Cancer Res 1982; 42: 4394–4398. 6290036

[bib44] Choong L-Y , Lim S , MC-S Loh , Man X , Chen Y , Toy W et al. Progressive loss of epidermal growth factor receptor in a subpopulation of breast cancers: implications in target-directed therapeutics. Mol Cancer Ther 2007; 6: 2828–2842. 17989321 10.1158/1535-7163.MCT-06-0809

[bib45] Strickland LB , Dawson PJ , Santner SJ , Miller FR . Progression of premalignant MCF10AT generates heterogeneous malignant variants with characteristic histologic types and immunohistochemical markers. Breast Cancer Res Treat 2000; 64: 235–240. 11200773 10.1023/a:1026562720218

[bib46] Morris VL , Tuck AB , Wilson SM , Percy D , Chambers AF . Tumor progression and metastasis in murine D2 hyperplastic alveolar nodule mammary tumor cell lines. Clin Exp Metastasis 1993; 11: 103–112. 8422701 10.1007/BF00880071

[bib47] Wendt MK , Taylor MA , Schiemann BJ , Schiemann WP . Down-regulation of epithelial cadherin is required to initiate metastatic outgrowth of breast cancer. Mol Biol Cell 2011; 22: 2423–2435. 21613543 10.1091/mbc.E11-04-0306 PMC3135469

[bib48] Dittadi R , Donisi PM , Brazzale A , Cappellozza L , Bruscagnin G , Gion M . Epidermal growth factor receptor in breast cancer. Comparison with non malignant breast tissue. Br J Cancer 1993; 67: 7–9. 8427782 10.1038/bjc.1993.2 PMC1968236

[bib49] Scartozzi M , Bearzi I , Mandolesi A , Giampieri R , Faloppi L , Galizia E et al. Epidermal growth factor receptor (EGFR) gene promoter methylation and cetuximab treatment in colorectal cancer patients. Br J Cancer 2011; 104: 1786–1790. 21559018 10.1038/bjc.2011.161 PMC3111171

[bib50] Wang Y-N , Lee H-H , Lee H-J , Du Y , Yamaguchi H , Hung M-C . Membrane-bound trafficking regulates nuclear transport of integral epidermal growth factor receptor (EGFR) and ErbB-2. J Biol Chem 2012; 287: 16869–16879. 22451678 10.1074/jbc.M111.314799 PMC3351284

[bib51] Brand TM , Iida M , Li C , Wheeler DL . The nuclear epidermal growth factor receptor signaling network and its role in cancer. Discov Med 2011; 12: 419–432. 22127113 PMC3305885

[bib52] Wang Y-N , Wang H , Yamaguchi H , Lee H-J , Lee H-H , Hung M-C . COPI-mediated retrograde trafficking from the Golgi to the ER regulates EGFR nuclear transport. Biochem Biophys Res Commun 2010; 399: 498–504. 20674546 10.1016/j.bbrc.2010.07.096 PMC2935258

[bib53] Lin SY , Makino K , Xia W , Matin A , Wen Y , Kwong KY et al. Nuclear localization of EGF receptor and its potential new role as a transcription factor. Nat Cell Biol 2001; 3: 802–808. 11533659 10.1038/ncb0901-802

[bib54] Li C , Iida M , Dunn EF , Ghia AJ , Wheeler DL . Nuclear EGFR contributes to acquired resistance to cetuximab. Oncogene 2009; 28: 3801–3813. 19684613 10.1038/onc.2009.234 PMC2900381

[bib55] Huang W-C , Chen Y-J , Li L-Y , Wei Y-L , Hsu S-C , Tsai S-L et al. Nuclear translocation of epidermal growth factor receptor by Akt-dependent phosphorylation enhances breast cancer-resistant protein expression in gefitinib-resistant cells. J Biol Chem 2011; 286: 20558–20568. 21487020 10.1074/jbc.M111.240796 PMC3121497

[bib56] Brand TM , Iida M , Dunn EF , Luthar N , Kostopoulos KT , Corrigan KL et al. Nuclear epidermal growth factor receptor is a functional molecular target in triple-negative breast cancer. Mol Cancer Ther 2014; 13: 1356–1368. 24634415 10.1158/1535-7163.MCT-13-1021 PMC4013210

[bib57] Yu Y-L , Chou R-H , Liang J-H , Chang W-J , Su K-J , Tseng Y-J et al. Targeting the EGFR/PCNA signaling suppresses tumor growth of triple-negative breast cancer cells with cell-penetrating PCNA peptides. PloS One 2013; 8: e61362. 23593472 10.1371/journal.pone.0061362 PMC3620387

[bib58] Schonbrunn A , Krasnoff M , Westendorf JM , Tashjian AJ . Epidermal growth factor and thyrotropin-releasing hormone act similarly on a clonal pituitary cell strain. Modulation of hormone production and inhibition of cell proliferation. J Cell Biol 1980; 85: 786–797. 6771298 10.1083/jcb.85.3.786 PMC2111439

[bib59] Gill GN , Lazar CS . Increased phosphotyrosine content and inhibition of proliferation in EGF-treated A431 cells. Nature 1981; 293: 305–307. 6268987 10.1038/293305a0

[bib60] Barnes DW . Epidermal growth factor inhibits growth of A431 human epidermoid carcinoma in serum-free cell culture. J Cell Biol 1982; 93: 1–4. 7040412 10.1083/jcb.93.1.1 PMC2112100

[bib61] Filmus J , Pollak MN , Cailleau R , Buick RN . MDA-468, a human breast cancer cell line with a high number of epidermal growth factor (EGF) receptors, has an amplified EGF receptor gene and is growth inhibited by EGF. Biochem Biophys Res Commun 1985; 128: 898–905. 2986629 10.1016/0006-291x(85)90131-7

[bib62] Prasad KA , Church JG . EGF-dependent growth inhibition in MDA-468 human breast cancer cells is characterized by late G1 arrest and altered gene expression. Exp Cell Res 1991; 195: 20–26. 1675999 10.1016/0014-4827(91)90495-g

[bib63] Kawamoto T , Mendelsohn J , Le A , Sato GH , Lazar CS , Gill GN . Relation of epidermal growth factor receptor concentration to growth of human epidermoid carcinoma A431 cells. J Biol Chem 1984; 259: 7761–7766. 6330080

[bib64] Choi J , Moon SY , Hong JP , Song J , Oh KT , Lee S . Epidermal growth factor induces cell death in the absence of overexpressed epidermal growth factor receptor and ErbB2 in various human cancer cell lines. Cancer Invest 2010; 28: 505–514. 20073577 10.3109/07357900902783179

[bib65] Carpenter G , Cohen S . Epidermal Growth Factor. Annu Rev Biochem 1979; 48: 193–216. 382984 10.1146/annurev.bi.48.070179.001205

[bib66] Hyatt DC , Ceresa BP . Cellular localization of the activated EGFR determines its effect on cell growth in MDA-MB-468 cells. Exp Cell Res 2008; 314: 3415–3425. 18817771 10.1016/j.yexcr.2008.08.020 PMC2590673

[bib67] Rush JS , Quinalty LM , Engelman L , Sherry DM , Ceresa BP . Endosomal accumulation of the activated epidermal growth factor receptor (EGFR) induces apoptosis. J Biol Chem 2012; 287: 712–722. 22102283 10.1074/jbc.M111.294470 PMC3249126

[bib68] Chin YE , Kitagawa M , Su WC , You ZH , Iwamoto Y , Fu XY . Cell growth arrest and induction of cyclin-dependent kinase inhibitor p21 WAF1/CIP1 mediated by STAT1. Science 1996; 272: 719–722. 8614832 10.1126/science.272.5262.719

[bib69] Kottke TJ , Blajeski AL , Martins LM , Mesner PWJ , Davidson NE , Earnshaw WC et al. Comparison of paclitaxel-, 5-fluoro-2’-deoxyuridine-, and epidermal growth factor (EGF)-induced apoptosis. Evidence for EGF-induced anoikis. J Biol Chem 1999; 274: 15927–15936. 10336499 10.1074/jbc.274.22.15927

[bib70] Andersen P , Pedersen MW , Woetmann A , Villingshøj M , Stockhausen MT , Odum N et al. EGFR induces expression of IRF-1 via STAT1 and STAT3 activation leading to growth arrest of human cancer cells. Int J Cancer 2008; 122: 342–349. 17918184 10.1002/ijc.23109

[bib71] Grudinkin PS , Zenin VV , Kropotov AV , Dorosh VN , Nikolsky NN . EGF-induced apoptosis in A431 cells is dependent on STAT1, but not on STAT3. Eur J Cell Biol 2007; 86: 591–603. 17646016 10.1016/j.ejcb.2007.05.009

[bib72] Kozyulina PY , Okorokova LS , Nikolsky NN , Grudinkin PS . p38 MAP kinase enhances EGF-induced apoptosis in A431 carcinoma cells by promoting tyrosine phosphorylation of STAT1. Biochem Biophys Res Commun 2013; 430: 331–335. 23178573 10.1016/j.bbrc.2012.11.041

[bib73] Ohtsubo M , Gamou S , Shimizu N . Antisense oligonucleotide of WAF1 gene prevents EGF-induced cell-cycle arrest in A431 cells. Oncogene 1998; 16: 797–802. 9488044 10.1038/sj.onc.1201588

[bib74] Zhang Y , Pan T , Zhong X , Cheng C . Resistance to cetuximab in EGFR-overexpressing esophageal squamous cell carcinoma xenografts due to FGFR2 amplification and overexpression. J Pharmacol Sci 2014; 126: 77–83. 25242085 10.1254/jphs.14150fp

[bib75] Engelman JA , Zejnullahu K , Mitsudomi T , Song Y , Hyland C , Park JO et al. MET amplification leads to gefitinib resistance in lung cancer by activating ERBB3 signaling. Science 2007; 316: 1039–1043. 17463250 10.1126/science.1141478

[bib76] Byers LA , Diao L , Wang J , Saintigny P , Girard L , Peyton M et al. An epithelial-mesenchymal transition gene signature predicts resistance to EGFR and PI3K inhibitors and identifies Axl as a therapeutic target for overcoming EGFR inhibitor resistance. Clin Cancer Res 2013; 19: 279–290. 23091115 10.1158/1078-0432.CCR-12-1558 PMC3567921

[bib77] Meyer AS , Miller MA , Gertler FB , Lauffenburger DA . The receptor AXL diversifies EGFR signaling and limits the response to EGFR-targeted inhibitors in triple-negative breast cancer cells. Sci Signal 2013; 6: ra66. 23921085 10.1126/scisignal.2004155 PMC3947921

[bib78] Brown W , Tan L , Smith A , Gray NS , Wendt M . Covalent targeting of fibroblast growth factor receptor inhibits metastatic breast cancer. Mol Cancer Ther 2016; 15: 2096–2106. 27371729 10.1158/1535-7163.MCT-16-0136 PMC5010989

[bib79] Brown W , Akhand S , Wendt M . FGFR signaling maintains a drug persistant cell population following epitheial-mesenchymal transistion. Oncotarget 2016; 7:83424–83436. 27825137 10.18632/oncotarget.13117 PMC5347779

[bib80] Stuhlmiller TJ , Miller SM , Zawistowski JS , Nakamura K , Beltran AS , Duncan JS et al. Inhibition of lapatinib-induced kinome reprogramming in ERBB2-positive breast cancer by targeting BET family bromodomains. Cell Rep 2015; 11: 390–404. 25865888 10.1016/j.celrep.2015.03.037 PMC4408261

[bib81] Welte T , Kim IS , Tian L , Gao X , Wang H , Li J et al. Oncogenic mTOR signalling recruits myeloid-derived suppressor cells to promote tumour initiation. Nat Cell Biol 2016; 18: 632–644. 27183469 10.1038/ncb3355 PMC4884142

[bib82] Seguin L , Kato S , Franovic A , Camargo MF , Lesperance J , Elliott KC et al. An integrin β₃-KRAS-RalB complex drives tumour stemness and resistance to EGFR inhibition. Nat Cell Biol 2014; 16: 457–468. 24747441 10.1038/ncb2953 PMC4105198

[bib83] Brünner N , Spang-Thomsen M , Vindeløv L , Nielsen A . Effect of 17 beta-oestradiol on growth curves and flow cytometric DNA distribution of two human breast carcinomas grown in nude mice. Br J Cancer 1983; 47: 641–647. 6849802 10.1038/bjc.1983.102 PMC2011400

[bib84] Brünner N , Spang-Thomsen M , Vindeløv L , Nielsen A , Engelholm SA , Svenstrup B . Dose-dependent effect of 17 beta-estradiol determined by growth curves and flow cytometric DNA analysis of a human breast carcinoma (T61) grown in nude mice. Exp Cell Biol 1985; 53: 220–232. 4029478 10.1159/000163315

[bib85] Lønning PE , Taylor PD , Anker G , Iddon J , Wie L , Jørgensen LM et al. High-dose estrogen treatment in postmenopausal breast cancer patients heavily exposed to endocrine therapy. Breast Cancer Res Treat 2001; 67: 111–116. 11519859 10.1023/a:1010619225209

[bib86] Mahtani RL , Stein A , Vogel CL . High-dose estrogen as salvage hormonal therapy for highly refractory metastatic breast cancer: a retrospective chart review. Clin Ther 2009; 31 Pt 2: 2371–2378. 20110046 10.1016/j.clinthera.2009.11.002

[bib87] Lim YJ , Jeon S-R , Koh JM , Wu H-G . Tumor growth suppression and enhanced radioresponse by an exogenous epidermal growth factor in mouse xenograft models with A431 cells. Cancer Res Treat 2015; 47: 921–930. 25600061 10.4143/crt.2014.153 PMC4614224

